# Long-term excessive application of K_2_SO_4_ fertilizer alters bacterial community and functional pathway of tobacco-planting soil

**DOI:** 10.3389/fpls.2022.1005303

**Published:** 2022-09-28

**Authors:** Ya Lu, Ping Cong, Shuai Kuang, Lina Tang, Yuyi Li, Jianxin Dong, Wenjing Song

**Affiliations:** ^1^ Key Laboratory of Tobacco Biology and Processing, Ministry of Agriculture, Tobacco Research Institute of Chinese Academy of Agricultural Sciences, Qingdao, China; ^2^ Tobacco Science Research Institute, Fujian Tobacco Monopoly Administration, Fuzhou, China; ^3^ Institute of Agricultural Resources and Regional Planning, Chinese Academy of Agricultural Sciences, Beijing, China

**Keywords:** tobacco-planting soil, K_2_SO_4_ fertilizer, soil physicochemical properties, bacterial community, pathway

## Abstract

To improve tobacco leaf quality, excessive K_2_SO_4_ fertilizers were applied to soils in major tobacco-planting areas in China. However, the effects of K_2_SO_4_ application on soil microbial community and functions are still unclear. An eight-year field experiment with three kinds of K_2_SO_4_ amounts (low amount, K_2_O 82.57 kg hm^-2^, LK; moderate amount, K_2_O 165.07 kg hm^-2^, MK; high amount, K_2_O 247.58 kg hm^-2^, HK) was established to assess the effects of K_2_SO_4_ application on the chemical and bacterial characteristics of tobacco-planting soil using 16S rRNA gene and metagenomic sequencing approaches. Results showed that HK led to lower pH and higher nitrogen (N), potassium (K), sulfur(S) and organic matter contents of the soil than LK. The bacterial community composition of HK was significantly different from those of MK and LK, while these of MK and LK were similar. Compared to LK, HK increased the relative abundance of predicted copiotrophic groups (e.g. *Burkholderiaceae*, *Rhodospirillaceae* families and *Ellin6067* genus) and potentially beneficial bacteria (e.g. *Gemmatimonadetes* phylum and *Bacillus* genus) associated with pathogens and heavy metal resistance, N fixation, dissolution of phosphorus and K. While some oligotrophic taxa (e.g. *Acidobacteria* phylum) related to carbon, N metabolism exhibited adverse responses to HK. Metagenomic analysis suggested that the improvement of pathways related to carbohydrate metabolism and genetic information processing by HK might be the self-protection mechanism of microorganisms against environmental stress. Besides, the redundancy analysis and variation partitioning analysis showed that soil pH, available K and S were the primary soil factors in shifting the bacterial community and KEGG pathways. This study provides a clear understanding of the responses of soil microbial communities and potential functions to excessive application of K_2_SO_4_ in tobacco-planting soil.

## Introduction

Tobacco (*Nicotiana tobacum* L.) is an economically important crop that is widely planted worldwide. As a main ingredient in tobacco, K is recognized as an important indicator of quality by the cigarette industry. A higher K content in tobacco leaves can improve the flammability, aroma and processability of cigarettes (Zhang and Kong, 2014). K plays a crucial role in cell expansion, the transportation of compounds, stomatal opening and closing and the activation of enzymes, which could promote the growth of tobacco (Hu et al., 2019). China is a main producing country of flue-cured tobacco in the world, while K deficiency of soil is a common problem in major tobacco-producing areas. As a result, the K content in tobacco is generally lower than the global standard for high-quality tobacco (Ding et al., 2017). K_2_SO_4_ is a high-quality and efficient K fertilizer with good solubility and a lack of chlorine, and the application of K_2_SO_4_ is currently the most effective measure to improve soil K content and tobacco quality. However, to pursue higher tobacco quality, a large amount of K_2_SO_4_ fertilizer (270-360 kg hm^-2^) is often applied to tobacco-planting soil in China. The application rate of K fertilizer is far beyond the K requirement of tobacco plants, leading to a large amount of K^+^ and ${\text{SO}}_{4}^{2-}$ left in the soil. Moreover, long-term application of K_2_SO_4_ possibly increased acidification in acidic and neutral soils, mainly due to the H^+^ released by tobacco roots when it absorbed excess K^+^ from the soil (Dai et al., 2021). These changes would lead to loss of base ions and inhibition of microbial activity and tobacco root growth (Shen et al., 2018). Therefore, environmental degradation caused by long-term excessive application of K_2_SO_4_ greatly limits the sustainable development of tobacco industry, which should be of concern.

Soil microbes are vital for maintaining soil quality and ecosystem, including the turnover of organic matter (OM), the degradation of toxic substances, the acceleration of nutrient availability and the improvement of stress tolerance to pathogens (Jin et al., 2022a). At the same time, soil microbes are also closely related to nutrient uptake, disease occurrence, growth and quality of tobacco (Zheng et al., 2021; Jin et al., 2022b). The variety and quantity of microbes in tobacco-planting soil are abundant. For example, *Proteobacteria*, *Actinobacteria*, *Acidobacteria*, *Firmicutes* and *Bacteroidetes* were always the dominant phyla in tobacco-planting soil (Zheng et al., 2021). *Arthrobacter* and *Lysobacter* were reported to be significant negative correlated with tobacco bacterial wilt disease (She et al., 2017). The increase of *Codinaea acaciae* and *Saitozyma podzolica* species were adverse to tobacco nicotine (Wang et al., 2022). Fertilization is an important means of shaping soil microorganisms. While excessive fertilization leads to the deterioration of soil physicochemical properties and microbial diversity and communities, which in turn leads to a decrease in tobacco yield and quality (Xin et al., 2012). For instance, excessive K fertilizer delays ripening and limits nicotine production (Henry et al., 2019).

Previously, pH and contents of OM, AK and water were considered to be key environmental factors, which significantly shaped the microbial community and diversity of tobacco-planting soils (Wang Z. B. et al., 2019; Shen et al., 2022). Excessive application of K_2_SO_4_ reduced the pH, which is widely recognized as the strongest predictor of microbial activity and composition (Rath et al., 2019; Zeng et al., 2019) and explain approximately 70% of species changes (Liu et al., 2022). Previous studies showed that soil pH had significantly positive relationships with soil bacterial α-diversity and bacterial operational taxonomic unit abundance and influenced ecological functions and biogeographic distribution (Wang C. et al., 2019). Wan et al. (2020) demonstrated that enzymes and proteins related to carbon (C), N, phosphorus (P) and S were downregulated in more acidic soils (pH< 5.5) compared to those in soils with pH values higher than 5.5. Wang W. et al. (2019) found that with decreasing pH, the functions of nitrification, ammonia oxidation, N fixation, nitrite respiration, and denitrification were restrained, while the functions of chemoheterotrophy, nitrate reduction and aromatic compound degradation were enriched.

A large amount of residual ${\text{SO}}_{4}^{2-}$ in soil not only increases soil acidification but also activates Fe^3+^ and Al^3+^ in soil and forms precipitation with Ca^2+^ and Mg^2+^ (Xu and Ji, 2001). Under anaerobic conditions, excess ${\text{SO}}_{4}^{2-}$ in soil may form H_2_S, which destroys aerobic beneficial microorganisms and promotes anaerobic harmful microorganisms (Liu et al., 2021). ${\text{SO}}_{4}^{2-}$ addition has been shown to significantly affect soil microbial communities by enriching specific microbial taxa associated with the bioavailability and transformation of metals such cadmium, arsenic and iron (Fe) (Li et al., 2019; Wang et al., 2021). Moreover, the addition of Na_2_SO_4_ altered the bacterial composition of the dominant phyla by increasing the relative abundance of *Proteobacteria* and *Acidobacteria* and depleting *Firmicutes* (Tang et al., 2020).

Abundant K^+^ in soil has an antagonistic effect with Ca^2+^, ${\text{NH}}_{4}^{+}$ and so on (Nieder et al., 2011), leading to nutrient imbalance in tobacco soils and plants. Twenty-one consecutive years of KCl application alone significantly decreased the Shannon, Simpson and McIntosh indices of the functional diversity of microbial communities compared to no fertilizer treatment in maize soil (Zhong et al., 2010).

However, studies on the regulation of soil chemical and biological characteristics by K and S fertilizers mostly focus on fertilizers such as KCl and Na_2_SO_4_. Moreover, many previous studies have shown that a single application of N fertilizer causes soil acidification and indirectly affects soil microbial communities (Bai et al., 2020; Yang et al., 2020). However, the effects of K_2_SO_4_ application and its resulting acidification on soil biological properties are still unclear, especially in tobacco fields. Therefore, in this study, we applied 16S rRNA gene and metagenomic sequencing technologies to analyze changes in the microbial community structure and function of tobacco-planting soil treated with three K_2_SO_4_ rates (LK, MK and HK) based on an 8-year experiment. We hypothesized that, HK had a negative and strong impact on the soil bacterial community and functions, when compared to LK. And MK had less effect on soil biological properties than HK. The variations of biological properties were due to the altered soil physicochemical properties, especially soil pH. The objectives of this study were to investigate (1) the effects of 8-year application of K_2_SO_4_ on soil physicochemical properties and nutrient uptake of tobacco plants; (2) the effects of 8-year application of K_2_SO_4_ on soil bacterial diversity and community composition of tobacco-planting soil; (3) impacts of 8-year application of K_2_SO_4_ on potential functional pathways of tobacco-planting soil; and (4) the key soil environmental variables that strongly affected soil microbial community and function.

## Materials and methods

### Site description and experimental design

This study was established in tobacco resources and environment field scientific observation and experiment station of the Chinese Academy of Agricultural Sciences (36°26’54″N, 120°34’38″E, 75 m a.s.l.) in 2010 in Qingdao city, Shandong province, China. This region has a temperate monsoon climate with a mean annual rainfall of 708.9 mm, average daily air temperature of 12.1°C, frost-free period of 200 d and annual accumulated temperature of 4410°C. The typical soil in this region is Alfisols (FAO Soil Taxonomic System). Before the experiment began in May 2010, the soil at 0-20 cm depth had a pH of 5.56, OM of 11.66 g kg^-1^, AN, available phosphorus (AP) and AK of 52.69 mg kg^-1^, 10.60 mg kg^-1^ and 105.25 mg kg^-1^, respectively.

Three treatments were included in the experiment: (1) applying compound fertilizer (N 15%, P_2_O_5_ 15%, K_2_O 15%) 550.5 kg hm^-2^ (LK); (2) applying compound fertilizer 550.5 kg hm^-2^ and K_2_SO_4_ (K_2_O 50%, S 18%) 165 kg hm^-2^ (MK); and (3) applying compound fertilizer 550.5 kg hm^-2^ and K_2_SO_4_ 330 _kg_ hm^-2^ (HK). The nutrient application amount of each treatment is shown in **Table 1**. The experiment used a randomized block design. Each plot (5 m long and 4.4 m wide) was separated by concrete walls from others. Each treatment had 3 replicates, giving a total of 9 pots. The variety used in the experiment was NC89, and 40 tobacco plants were planted in each plot with a line spacing of 1.1 m and row of 0.5 m. All fertilizers were applied to the surface in a certain amount, and then mixing with 0-10 cm soil manually, ridging and planting tobacco. The tobaccos were transplanted in the first ten days of June every year. Irrigation was carried out according to the water requirement of tobacco and rainfall during different growth periods. The other field management practices were in accordance with local farming practices.

**Table 1:** Nutrient application rate of each treatment.

Treatments	NPK ratio	Nutrient input (kg hm^-2^)			
		N	P_2_O_5_	K_2_O	S
LK	1:1:1	82.57	82.57	82.57	66.06
MK	1:1:2	82.57	82.57	165.07	95.76
HK	1:1:3	82.57	82.57	247.57	125.46

LK, Low amount of K_2_SO_4_; MK, Middle amount of K_2_SO_4_; HK, High amount of K_2_SO_4_.

### Sampling

Soil and plant samplings were conducted in the mature season of flue-cured tobacco on August 30, 2017. One composite rhizosphere soil sample was taken from each plot consisting of roots of 5 randomly selected tobacco plants. The roots were uprooted by shaking the roots, removing the loose soil at the roots, and collecting the soil at the roots with a sterile brush. Then, the fresh soil was passed through a 2 mm sieve and divided into two fractions. One part of the fresh soil was used for the analysis of bacterial community and functions. The other part was air dried for the determination of soil physicochemical properties. After collecting soil, the 5 tobacco plants taken from each plot were washed and then were mixed into one sample for determining dry matter weight and nutrient content. Three composite samples of both soil and plant were conducted for each treatment.

### Analysis of soil physicochemical properties and plant indices

Soil physicochemical properties and plant nutrients were determined according to Bao (2011). Soil pH was measured with a soil-to-water ratio of 1:2.5 using a pH meter (Meter3100C the US). The OM content was analyzed using dichromate oxidation. The total nitrogen (TN) content was digested by H_2_SO_4_-K_2_Cr_2_O_7_ and measured by Kjeldahl digestion with automatic N analyzer (KjeltecTM 8400 Denmark). The AN content was determined by the alkali-diffusion method. Total potassium (TK) and AK were digested by HClO_4_ and CH_3_COONH_4_, respectively, and both were measured by flame atomic absorption spectrophotometry with flame spectrometry (Sherwood M410 Britain). Total sulfur (TS) and AS were digested by Mg(NO_3_)_2_ and extracting agent of Ca(H_2_PO_4_)_2_ and CH_3_COOH, respectively, and both were determinated by BaSO_4_ turbidimetry with UV-visible spectrophotometer (SHIMADZU UV-2700 Japan).

The plant dry matter was weighed after drying at 105°C for 30 mins and subsequently at 80°C to a constant weight. Then, the dry plant was ground and sieved to< 2 mm. The contents of plant N and K were digested by H_2_SO_4_-H_2_O_2_ digestion and then N content was determined by the Kjeldahl method with automatic N analyzer and K content was measured by flame photometry with flame spectrometry. The contents of plant S were digested by HNO_3_-HClO_4_ and then measured by the BaSO_4_ turbidimetric method with UV-visible spectrophotometer. N (K, S) accumulation was calculated as the product of dry matter and the N (K, S) content of the plant.

### 16S rRNA gene sequencing and analysis

Soil DNA was extracted using a PowerSoil DNA Isolation Kit (MoBio Laboratories, Carlsbad, CA) following the manual. The purity and quality of the genomic DNA were checked on 0.8% agarose gels.

The V3-4 hypervariable region of the bacterial 16S rRNA gene was amplified with the primers 338F (5’-ACTCCTACGGGAGGCAGCAG-3’) and 806R (5’-GGACTACNNGGGTATCTAAT-3’) (Caporaso et al., 2012). For each soil sample, an 8-digit barcode sequence was added to the 5’ end of the forward and reverse primers (provided by Allwegene Company, Beijing). PCR was carried out on a Mastercycler Gradient (Eppendorf, Germany) using 25 μl reaction volumes, containing 12.5 μl 2× Taq PCR MasterMix, 3 μl BSA (2 ng μl^-1^), 1 μl forward primer (5 μM), 1 μL reverse primer (5 μM), 2 μl template DNA, and 5.5 μl ddH_2_O. The cycling parameters were 95°C for 5 min, followed by 28 cycles of 95°C for 45 s, 55°C for 50 s and 72°C for 45 s with a final extension at 72°C for 10 min. The PCR products were purified using an Agencourt AMPure XP Kit.

The raw data were first screened, and sequences were removed from consideration if they were shorter than 230 bp, had a low-quality score (≤ 20), contained ambiguous bases or did not exactly match to primer sequences and barcode tags and separated using the sample-specific barcode sequences. Qualified reads were clustered into operational taxonomic units (OTUs) at a similarity level of 97% using the Uparse algorithm of Vsearch (v2.7.1) software (Edgar, 2013). The Ribosomal Database Project (RDP) Classifier tool was used to classify all sequences into different taxonomic groups against the SILVA128 database (Cole et al., 2009). The sequencing was performed on Illumina Miseq PE300 platform. The raw sequences of the 16S rRNA gene were deposited into the NCBI database under the accession number PRJNA805374.

### Metagenome shotgun sequencing and analysis

Total microbial genomic DNA samples were extracted using the OMEGA Soil DNA Kit (D5625-01), following the manufacturer’s instructions, and stored at -20°C prior to further assessment. The quantity and quality of extracted DNAs were measured using a NanoDrop ND-1000 spectrophotometer (Thermo Fisher Scientific, Waltham, MA,USA) and agarose gel electrophoresis, respectively. The extracted microbial DNA was processed to construct metagenome shotgun sequencing libraries with insert sizes of 400 bp by using Illumina TruSeq Nano DNA LT Library Preparation Kit. Each library was sequenced by Illumina HiSeq X-ten platform (Illumina, USA) with PE150 strategy.

Raw sequencing reads were processed to obtain quality-filtered reads for further analysis. Firstly, sequencing adapters were removed from sequencing reads using Cutadapt (v1.2.1) (Martin, 2011). Secondly, low-quality reads were trimmed using a sliding-window algorithm in fastp (Chen et al., 2018). Megahit (v1.1.2) (Li et al., 2015) was used to assemble each sample using the meta-large preset parameters. The generated contigs (longer than 200 bp) were then pooled together and clustered using mmseqs2 (Steinegger and Soding, 2017) with “easy-Linclust” mode, setting the sequence identity threshold to 0.95 and covering residues of the shorter contig to 90%. MetaGeneMark (Zhu et al., 2010) was used to predict the genes in the contigs. The CDSs of all samples were clustered by mmseqs2 (Steinegger and Soding, 2017) with “easy-cluster” mode, setting the protein sequence identity threshold to 0.90 and covering residues of the shorter contig to 90%. To assess the abundances of these genes, the high-quality reads from each sample were mapped onto the predicted gene sequences using salmon (Patro et al., 2015) in the quasi-mapping-based mode with “–meta –minScoreFraction = 0.55”, and the CPM (copy per kilobase per million mapped reads) was used to normalize abundance values in metagenomes. The functionality of the nonredundant genes was obtained by annotation using mmseqs2 (Steinegger and Soding, 2017) with the “search” mode against the protein databases of KEGG.

### Statistical analyses

Analysis of variance was conducted to determine significant differences in indices of soil physicochemical characteristics, α-bacterial diversity, plant dry matter and nutrient accumulation. OTUs and pathways (Level 3) were analyzed by principal coordinate analyses (PCoA) based on Bray–Curtis distance by CANOCO 5.0. Permutational multivariate analysis of variance (PERMANOVA) was conducted using the adonis function (Vegan package, Rstudio) to evaluate similarities in the OTUs and pathways. Venn diagrams were constructed to show the number of shared OTUs by MOTHUR software (Schloss et al., 2011). *Post hoc* analysis by Stamp was used to compare the differences in the top 20 phyla and genera between different soil samples. Differentially abundant OTUs between groups were calculated using a moderate t-test, and the obtained *P* values were adjusted using the Benjamini–Hochberg correction method. Enriched OTUs were further visualized in volcano and heatmap plots using the Limma R package and heatmap.2 function R package. Linear discriminant analysis effect size (LEfSe) for detecting significant differences in KEGG pathways was performed on the online Galaxy platform (http://huttenhower.sph.harvard.edu/galaxy/) (Segata et al., 2011). Redundancy analysis (RDA) and variation partitioning analysis (VPA) were applied to clarify the influence of environmental factors on the microbial community and functional composition. RDA and VPA were implemented using R project Vegan package (version 2.5.3) and CANOCO 5.0, respectively. Co-occurrence network analysis was used to explore environmental factor-OTU and environmental factor-pathway interactions. Only top100 OTUs and pathways with coefficients>0.85 (or<-0.85) and FDR corrected *P* values<0.05 (Spearman’s correlation) were identified and established into a network. All statistical analyses were performed using igraph package in R (Csardi and Nepusz, 2006) and networks were constructed and visualized in Cytoscape v.3.8.0 (Shannon et al., 2003). Structural equation modeling (SEM) was performed by SPSS-AMOS to analyze hypothetical pathways to explain soil physicochemical characteristics, bacterial community and KEGG pathways effects on plant biomass. The model fit was assessed by a χ2-test, the comparative fit index (CFI) and the root square mean error of approximation (RMSEA). Mean values ± SE were reported here.

## Result

### Soil physicochemical characteristics and plant nutrient accumulation

**Figure 1:**
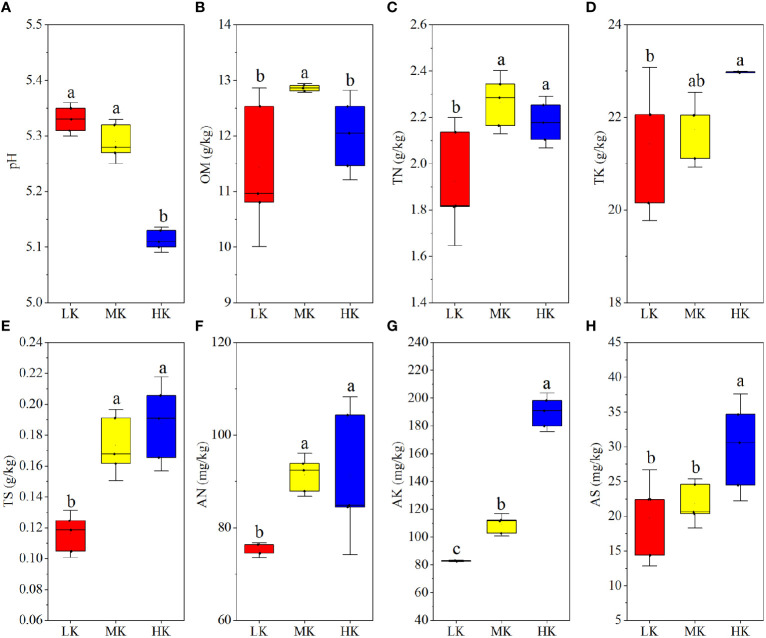
Soil physicochemical properties of plots with different fertilization regimes **(A-H)**. Different lowercase letters above the boxes indicate significant differences (P < 0.05) among the different treatments, as determined by ANOVA followed by the LSD test. LK, Low amount of K_2_SO_4_; MK, Middle amount of K_2_SO_4_; HK, High amount of K_2_SO_4_. OM, organic matter; TN, total nitrogen; TK, total potassium; TS, total sulfur; AN, available nitrogen; AK, available potassium; AS, available sulfur.

The results of a comparative analysis of soil physicochemical properties among different K_2_SO_4_ treatments are presented in **Figure 1**. Applying medium and high amounts of K_2_SO_4_ (MK and HK) promoted N (AN and TN), K (AK and TK) and S (AS and TS) contents compared to LK, and the differences between HK and LK was always remarkable. The OM contents were also higher in MK and HK than in LK and there was significant difference between MK and LK. Unlike other soil nutrients, pH dropped with the increasing K_2_SO_4_ rate, and the difference between LK and HK was significant.

**Figure 2:**
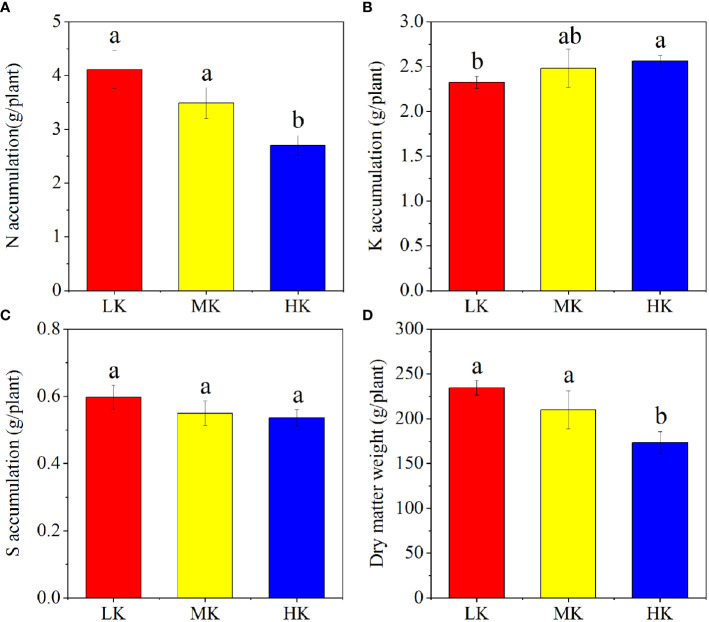
N **(A)**, K **(B)**, S **(C)** and dry matter **(D)** accumulation in plants under different treatments. Different lowercase letters above the bars indicate significant differences (P< 0.05) among the different treatments, as determined by ANOVA followed by the LSD test. LK, Low amount of K_2_SO_4_; MK, Middle amount of K_2_SO_4_; HK, High amount of K_2_SO_4_.

The N, K, S nutrient and dry matter accumulation of different plots are displayed in **Figure 2**. The N and dry matter accumulation of whole plant decreased with increasing K_2_SO_4_ amount, while the K accumulation showed the opposite trend. Compared to LK, the dry matter accumulation was decreased by 10.50% and 26.05% in MK and HK, respectively. The differences in accumulations of N, K and dry matter between LK and HK were all remarkable.

### α and β diversity of bacteria

Through the 16S rRNA gene sequencing analysis of 9 soil samples, a total of 36020-40641 raw tags and 34116-337892 clean tags were obtained (**Table 2**). The sequencing coverage rate was 0.9760-0.9778 with 97% similarity. The results basically covered all the species in the tested samples, and further analysis of the bacterial community structure could be carried out.

**Table 2:** Sequencing results of soil samples.

Treatments	16S			Metagenomics			
	Raw_tags	Clean tags	Goods coverage	ReadsCount	BasesCount (bp)	Q20 (%)	Q30 (%)
LK	40641	37892	0.9761	117919397	17687909500	97.81	94.38
MK	39884	37688	0.9760	107915497	16187324600	97.85	94.47
HK	36020	34116	0.9778	116254271	17438140600	97.83	94.37

LK, Low amount of K_2_SO_4_; MK, Middle amount of K_2_SO_4_; HK, High amount of K_2_SO_4_.

**Figure 3:**
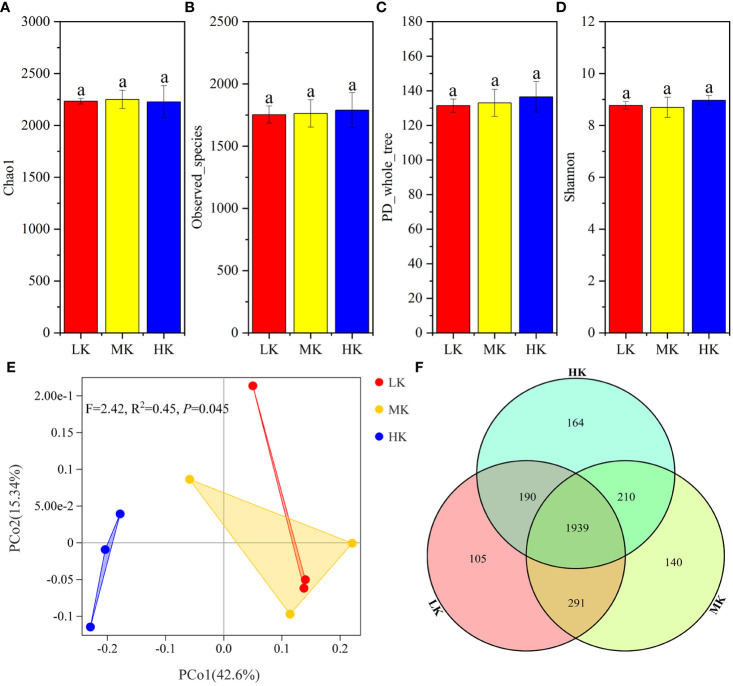
Similarity and differentiation of the bacterial community with different fertilization treatments. **(A-D)**. α-diversity of soils treated with different fertilization treatments; **(E)**. PCoA plot of bacterial communities at the OTU level; **(F)**. Venn diagram of exclusive and shared bacterial taxa at the OTU level. Values with the same lowercase letters are not significantly different among the different treatments (LSD test). LK, Low amount of K_2_SO_4_; MK, Middle amount of K_2_SO_4_; HK, High amount of K_2_SO_4_.

α-diversity analysis based on OTU showed that Chao1, Observed_species, PD_whole_tree and Shannon were not significantly different among the three treatments, signifying that the addition of K_2_SO_4_ had little effect on bacterial α-diversity (**Figure 3**).

PCoA is performed to determine the OTU compositions in soils with different treatments (**Figure 3**). PERMANOVA results showed that application of K_2_SO_4_ explained the variation in OTU compositions significantly (*P* =0.045). The variance contribution rates of the first and second principal components were 42.6% and 15.34%, respectively. The three treatments could be divided into two groups along the PCoA1 axis. The three samples for the HK gathered together on the left side of the abscissa PCoA1, well separated from those of MK and LK. Most samples of MK and LK were clustered together on the right half of the plot. It was indicated that the bacterial composition of MK and LK was similar, and HK significantly changed the bacterial community.

The Venn diagram (**Figure 3**) showed that the number of OTUs unique to each treatment increased from 105 to 164 with the increasing K_2_SO_4_ rate, and the number of OTUs shared by LK and MK (2230) was greater than that shared by HK and LK (2129) and HK and MK (2149). These sequencing data indicated that applying a high amount of K_2_SO_4_ had a stronger stimulatory effect on the soil bacterial community, but MK had little effect.

### Bacterial community composition

**Figure 4:**
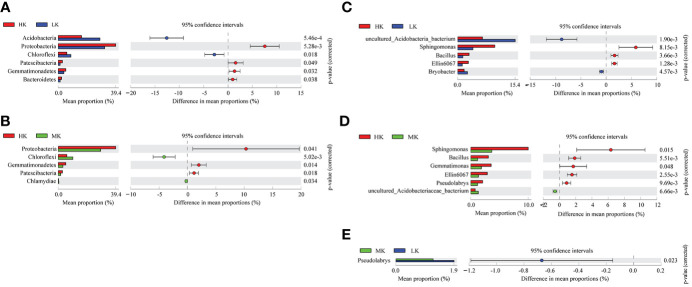
Comparative analysis of the top 20 species phyla and genera with significant differences between different treatments. **(A)** Differences at phylum level between LK and HK. **(B)** Differences at phylum level between LK and MK. **(C)** Differences at genus level between LK and HK. **(D)** Differences at genus level between MK and HK. **(E)** Differences at genus level between MK and LK. Only significant differences are shown (*P*< 0.05); LK, Low amount of K_2_SO_4_; MK, Middle amount of K_2_SO_4_; HK, High amount of K_2_SO_4_.

*Post hoc* tests are used to detect significant differences in the relative abundance of the top 20 phyla and genera (**Figure 4**). Addition of K_2_SO_4_ significantly altered the bacterial composition at the phylum and genus levels. HK enriched the relative abundances of *Proteobacteria*, *Gemmatimonadetes*, *Bacteroidetes*, *Patescibacteria* phyla and *Sphingomonas*, *Bacillus*, *Ellin6067* genera, while it reduced the proportions of *Acidobacteria*, *Chloroflexi* phyla and *uncultured_Acidobacteria_bacterium*, *Bryobacter* genera compared with LK. Only one genus, *Pseudolabrys*, showed significant difference between MK and LK, and there was no significant difference at the phylum level between the two treatments. This result indicated that HK greatly shaped the bacterial composition at the phylum and genus levels, while MK had little effect on the bacterial community.

**Figure 5:**
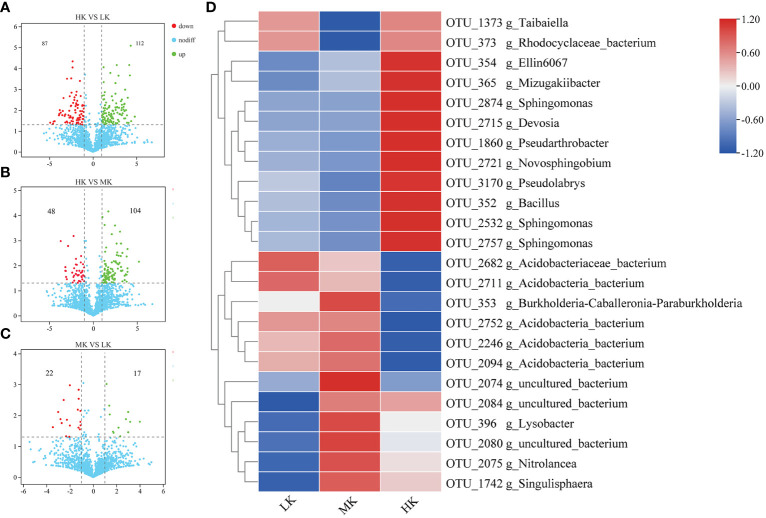
The relative abundance of enriched or depleted OTUs in different fertilized soils after pairwise comparison. **(A-C)** Volcano plot showing the differentially abundant OTUs between HK and LK, HK and MK, MK and LK. The position along the y-axis represents the log2 of average abundance of each OTU, and the x-axis represents the log2 of fold change between two groups. **(D)** Heatmap of the relative abundance of significantly enriched OTUs (top 30) among the different treatments.

We further identified OTUs correlated with the differences between different treatments to explore the enrichment or exclusion of different bacterial taxa by fertilization (**Figure 5**). The number of OTUs with significant differential relative abundance between the HK and LK group was greater than those between the HK and MK group and the MK and LK group, indicating that HK had a greater effect on the composition of OTUs than MK.

To display the differences in the relative abundance of bacterial operational taxonomic units (OTUs), a heatmap is constructed based on the top 30 enriched OTUs (**Figure 5**). Compared with LK, MK enriched 7 OTUs, and 4 of them belonged to uncultured_bacterium, and the other three were affiliated with *Lysobacter*, *Nitrolancea* and *Singulisphaera* genera, respectively. MK also depleted 3 OTUs belonging to *Taibaiella*, uncultured_bacterium and uncultured_*Rhodocyclaceae*. HK improved 5 OTUs compared to LK, and 3 of them were affiliated with *Sphingomonas* genus and the other two belonged to *Ellin6067* and *Pseudarthrobacter* genera, respectively. There were 5 OTUs downregulated in HK compared to LK. And 4 of them belonged to uncultured_*Acidobacteria*_bacterium and the other belonged to uncultured_bacterium.

### Potential functional pathways

**Figure 6:**
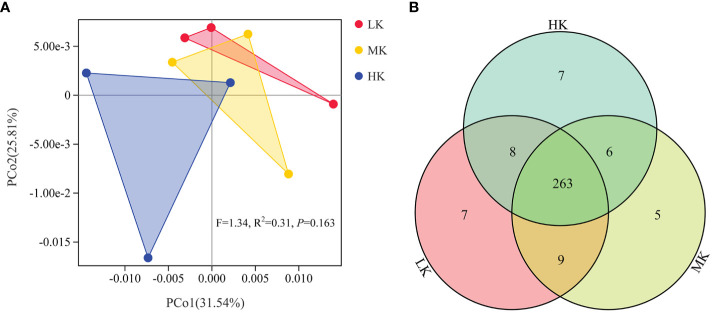
Similarity and differentiation of KEGG pathways with different fertilization treatments. **(A)** PCoA plot of KEGG pathways at level 3; **(B)** Venn diagram of exclusive and shared KEGG pathways at level 3. LK, Low amount of K_2_SO_4_; MK, Middle amount of K_2_SO_4_; HK, High amount of K_2_SO_4_.

Phylogenetic investigation of communities by reconstruction of unobserved states (PICRUSt) analysis predicts KEGG functional pathways (Level 3) associated with the metagenomes of the three treatments. PCoA analysis is carried out to investigate the effects of the treatments on the composition of KEGG pathways (**Figure 6**). PERMANOVA results confirmed that application of K_2_SO_4_ had no significant effect on the composition of KEGG pathways (*P* =0.163). The variance contribution rates of the first and second principal components were 31.54% and 25.81%, respectively. The three samples of HK could be completely separated from those of the LK treatment. In comparison, the samples of MK were closer to those of LK. This result indicated that HK had a greater effect on the potential functional composition of microorganisms than MK. A Venn diagram showed small differences in the number of shared and exclusive pathways across the three treatments (**Figure 6**).

**Figure 7:**
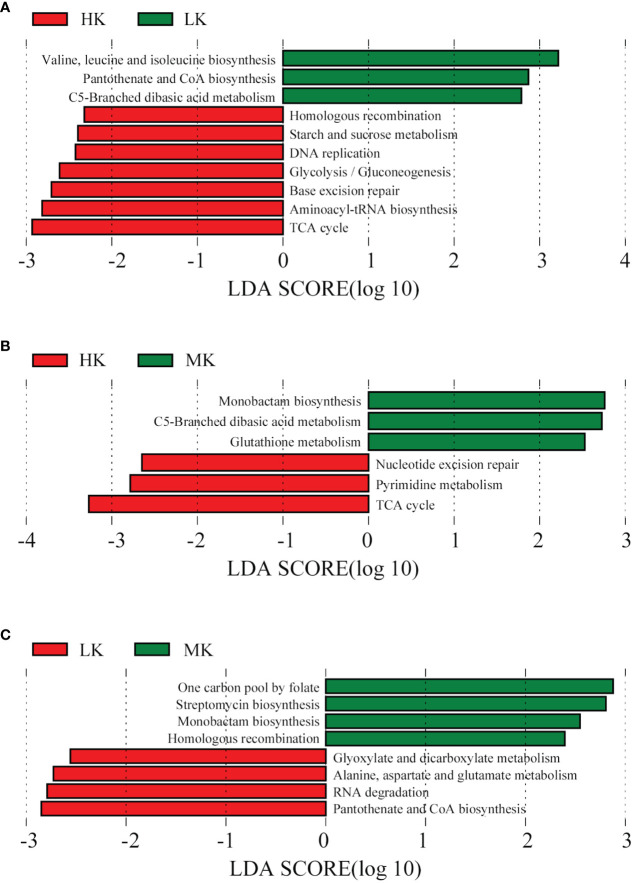
Histogram of the LDA effect value of differentially enriched KEGG pathways at level 3. Lineages with LDA values higher than 2.5 are displayed. **(A)** HK *vs*. LK; **(B)** HK *vs*. MK; **(C)**. MK *vs*. LK.

LEfSe analysis is conducted on the top 100 level 3 KEGG pathways to determine pathways at level 3 KEGG gene annotation with significant differences in abundance across the three treatments. When comparing HK and LK (**Figure 7**), HK was primarily associated with pathways of homologous recombination, starch and sucrose metabolism, DNA replication, glycolysis/gluconeogenesis, base excision repair, aminoacyl-tRNA biosynthesis and the TCA cycle, while LK was primarily associated with valine, leucine and isoleucine biosynthesis, pantothenate and CoA biosynthesis and C5-branched dibasic acid metabolism. In the MK and LK group (**Figure 7**), MK was mainly associated with the functions of one carbon pool by folate, streptomycin biosynthesis, monobactam biosynthesis and homologous recombination, while LK was mainly associated with glyoxylate and dicarboxylate metabolism, alanine, aspartate and glutamate metabolism, RNA degradation and pantothenate and CoA biosynthesis.

### Effects of physicochemical characteristics on bacterial community and functional pathways

**Figure 8:**
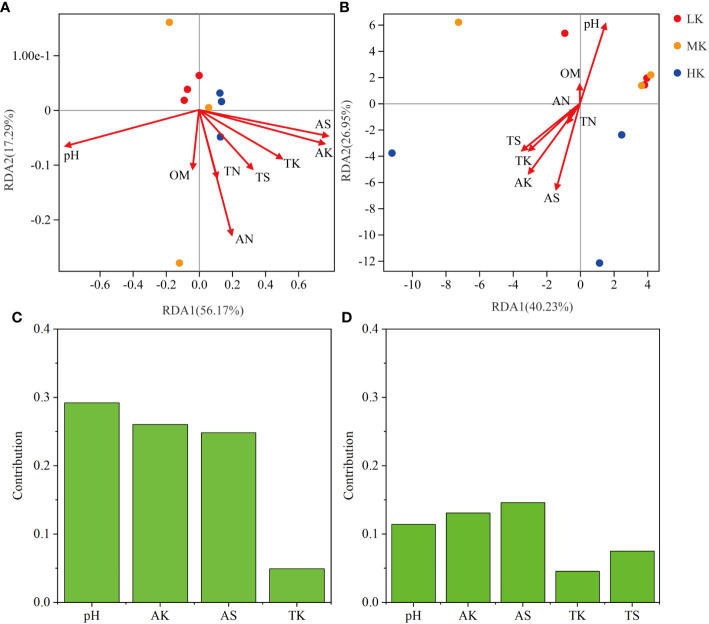
RDA analysis among soil samples based on all OTUs **(A)** and pathways_L3 **(B)**, and VPA analysis of the effects of soil physicochemical properties on OTUs **(C)** and pathways_L3 **(D)**. LK, Low amount of K_2_SO_4_; MK, Middle amount of K_2_SO_4_; HK, High amount of K_2_SO_4_. OM, organic matter; TN, total nitrogen; TK, total potassium; TS, total sulfur; AN, available nitrogen; AK, available potassium; AS, available sulfur.

The results of RDA analysis for the bacterial community and functional pathways are visualized in **Figure 8**. The first two axes explained 56.17% and 17.29% of the total variance in the bacterial community, respectively, and 40.23% and 26.95% in pathways. Among the 8 environmental variables, a remarkable impact of pH (r^2^ = 0.636, *P* = 0.029) was found in the bacterial community, as well as pH (r^2^ = 0.757, *P* = 0.025), AK (r^2^ = 0.74, *P* = 0.023) and AS (r^2^ = 0.878, *P* = 0.004) on pathway composition (**Table 3**). VPA analysis showed that pH, AK, AS and TK explained 29.23%, 26.03%, 24.84% and 4.95% of bacterial community variation, respectively (**Figure 8**). AS, AK, pH, TS and TK accounted for 14.61%, 13.09%, 11.42%, 7.53% and 4.57% of pathway variation, respectively (**Figure 8**). In summary, pH, AK and AS were the critical factors in shaping bacterial community and functional composition.

**Table 3:** Contribution of environmental factors to OTUs and pathways.

Environmental factors	OTUs		Pathways	
	*P* value	r^2^	*P* value	r^2^
pH OM TN	0.029 0.978 0.909	0.636 0.012 0.026	0.025 0.936 0.914	0.757 0.033 0.046
TK	0.416	0.25	0.167	0.428
TS	0.746	0.111	0.142	0.481
AN	0.781	0.089	0.933	0.022
AK	0.079	0.562	0.023	0.74
AS	0.086	0.593	0.004	0.878

OM, organic matter; TN, total nitrogen; TK, total potassium; TS, total sulfur; AN, available nitrogen; AK, available potassium; AS, available sulphur.

**Figure 9:**
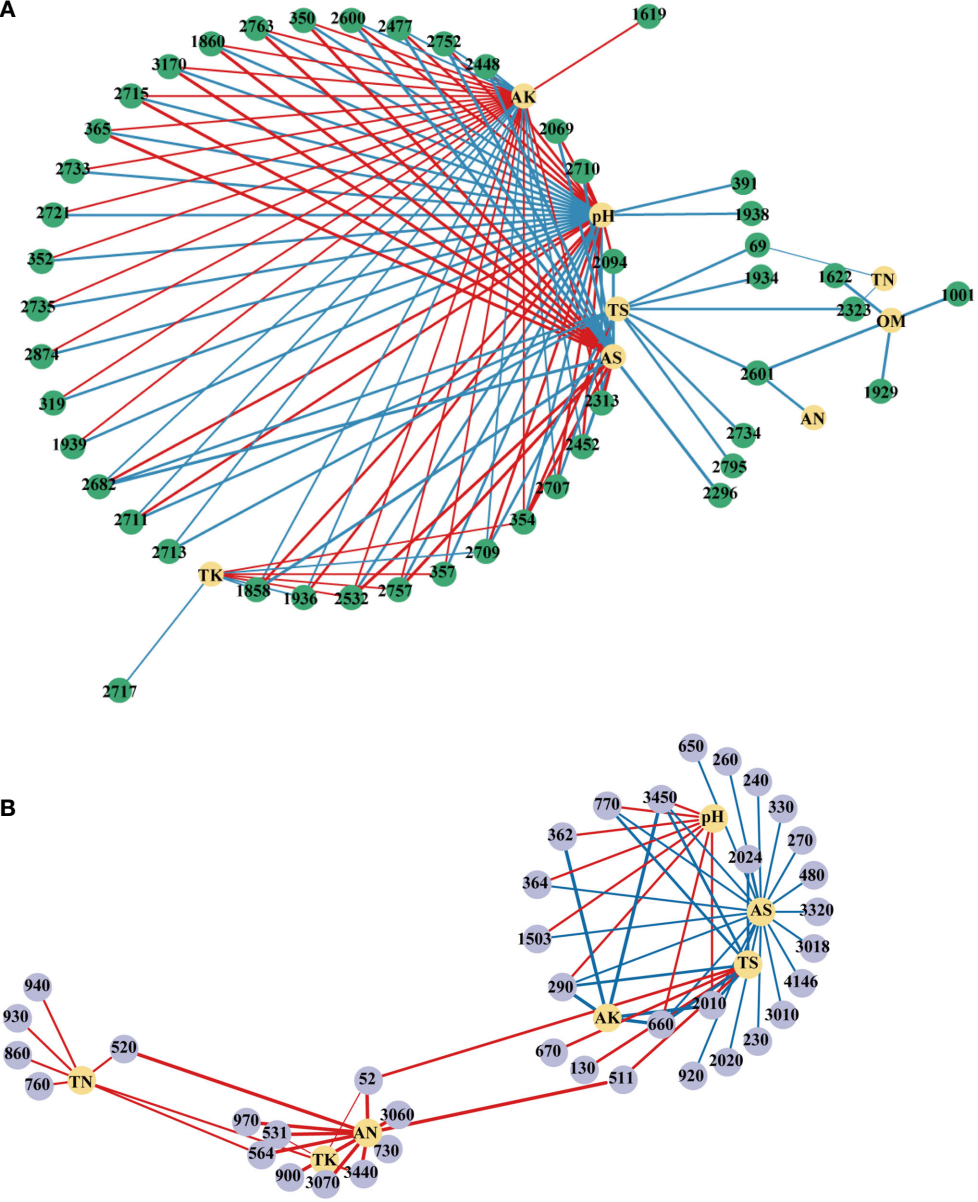
Network analysis revealing associations between soil physicochemical properties and top100 OTUs **(A)** and pathways **(B)**. A blue line indicates a negative interaction, while a red line indicates a positive interaction. The numbers in the figure represent the numbers of the OTU or pathway. LK, Low amount of K_2_SO_4_; MK, Middle amount of K_2_SO_4_; HK, High amount of K_2_SO_4_. OM, organic matter; TN, total nitrogen; TK, total potassium; TS, total sulfur; AN, available nitrogen; AK, available potassium; AS, available sulfur.

Further, the interactions between environmental factors and microbial communities and functions were investigated using person’s correlation and were visualized by co-occurrence networks (**Figure 9**). In the network of OTU, pH recorded the highest node connectivity (34), followed by AK (31) and AS (21). In most cases, pH had significantly or extremely significantly negative correlations with OTUs belonged to *Proteobacteria* and had positive correlations with OTUs belonged to *Acidobacteria* (**Table 1S**). AS and AK showed the opposite trends. In the network of pathways, AS recorded the highest node connectivity, and was significantly negatively correlated with 21 pathways, which belonged to pathways (L1) of Metabolism, Environmental Information Processing, Human Diseases, Genetic Information Processing, Cellular Processes, Organismal Systems (**Table 2S**). And the node connectivities of AN, TS and pH were 11, 10, and 8, respectively.

**Figure 10:**
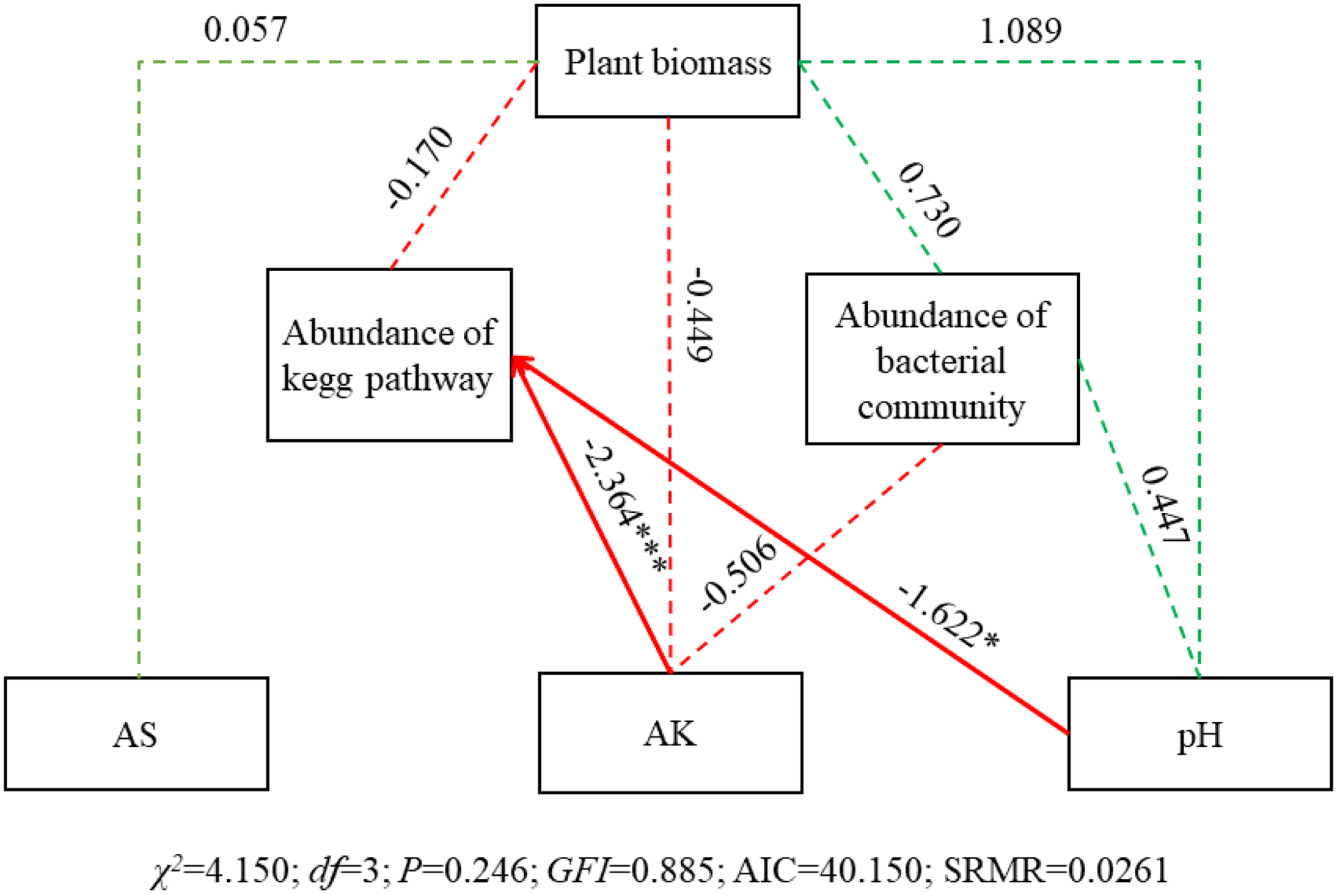
SEM of the effects of soil physicochemical characteristics, bacterial community and KEGG pathways on plant biomass. Square boxes denote variables included in the models. Values associated with solid arrows represent standardized path coefficients (SPCs) and asterisks mark their significance: *P< 0.05; ***P< 0.001. Solid arrows denote the directions and effects that were significant (*P*< 0.05). Dashed arrows represent the directions and effects that were non-significant (*P*< 0.05). Green arrows indicate a positive relationship (*P*< 0.05), while red indicates a negative correlation. CFI = 0.885 is result from the chi-squared value is less than the degrees of freedom in this SEM. Abundance of bacterial community, PCA1 of the abundance of the top 100 OTUs; Abundance of the abundance of the top 100 pathways; AK, available potassium; AS, available sulfur.

SEM results showed that AK and pH significantly affected abundance of KEGG pathway (SPC = -2.364, *P<* 0.001; SPC = -1.622, *P<* 0.05), while no significant effect on abundance of bacterial community (**Figure 10**). And all the three physicochemical indicators and abundance of bacterial community and KEGG pathway had no significant effect on plant biomass.

## Discussion

### Application of K_2_SO_4_ changed soil physicochemical properties and plant nutrient uptake

The results of the present study demonstrated that after the 8-year application of K_2_SO_4_ fertilizer significantly changed the physicochemical properties of tobacco-planting soil. MK and HK led to increase of soil acidification, when compared to LK (**Figure 1**). The main reasons for this phenomenon may be as follows. First, tobacco plant absorbed much more K^+^ than ${\text{SO}}_{4}^{2-}$ , and H^+^ was released by root to maintain the charge balance in its body (Wallace, 1994). Second, almost all the base ions (mainly K^+^) absorbed by tobacco plant were taken away from soil at the harvest time. When 1 mol of base ions were removed from the soil, the acid buffer capacity of the soil decreased by 1 mol (Dong et al., 2022). Third, to maintain the ion balance, when 1 mol of ${\text{SO}}_{4}^{2-}$ were leached from soil, the same amount of base ions would also be washed to maintain charge balance (Xu and Ji, 2001). Our results also showed that the AN contents were higher in MK and HK than that in LK (**Figure 1** C and F). It might be attributed to the following reasons. First, K application reduced soil N (mainly N_2_O emissions) loss by reducing denitrification (Li et al., 2021). Second, tobacco roots were damaged by the excess H^+^ and Al^3+^ brought by MK and HK (Dai et al., 2021), which inhibited the N uptake of plants (**Figure 2**) and left more N in the soil. Additionally, MK and LK improved OM contents (**Figure 1**), mainly because the increasing Fe and Al oxides of soil, caused by low pH, could protect OM by adsorbing organic biomolecules (Berhe et al., 2012). This finding was in accordance with previous studies (Wen et al., 2019; Huang et al., 2020), which reported that long-term application of only N fertilizer or combined application of NPK fertilizer improved soil OM content by reducing soil pH.

In our study, dry matter weight and N and S accumulation were restrained in the soil treated with HK (**Figure 2**). The main reason was that excessive application of K_2_SO_4_ intensified soil acidification, and the roots were harmed by the abundant H^+^ and Al^3+^ (Yang et al., 2018; Dai et al., 2021).

### Application of K_2_SO_4_ altered bacterial community and KEGG pathways

In our study, the percentages of *Proteobacteria*, *Gemmatimonadetes*, *Bacteroidetes*, *Patescibacteria* phyla and *Sphingomonas*, *Bacillus*, *Ellin6067* genera were significantly higher in HK than in LK (**Figure 4**). And except for *Bacillus*, most members of all these taxa are considered to be predicted copiotrophic bacteria (Xia et al., 2005; Spain et al., 2009; Albertsen et al., 2013; Mowlick et al., 2014; Naas et al., 2014; Brown et al., 2015; Banerjee et al., 2016), which grow fastly in nutrient-rich conditions (Fierer et al., 2007), such as *Burkholderiaceae*, *Rhodospirillaceae* and *Rhizobiaceae* families in *Proteobacteria* phylum (**Table 3S**; Finn et al., 2021). And all taxa (*Acidobacteria*, *Chloroflexi* phyla and *uncultured_Acidobacteria_bacterium*, *Bryobacter* genera) significantly reduced in HK were reported to be predicted oligotrophic bacteria (Sorokin et al., 2012; Dedysh et al., 2017; Kalam et al., 2020), which grow slowly and are able to metabolize nutrient poor and recalcitrant C substrates (Fierer et al., 2007). These shifts in community structure between HK and LK could be mainly ascribed to the more OM supplied by HK (**Figure 1B**). Interestingly, *Gemmatimonadetes* phyla, *Bacillus* and *Sphingomonas* genera enriched in the HK treatment were considered to be potential beneficial bacteria. *Gemmatimonadetes* facilitates P dissolution and suppress diseases (Zeng et al., 2020). *Bacillus* (belongs to *Firmicutes* phylum) has the abilities of resistance to pathogens, N fixation, dissolution of P and K (Radhakrishnan et al., 2017). *Sphingomonas* is reported to degrade recalcitrant compounds and fix N (Xu et al., 2018). It was mainly because soil acidification of HK increased harmful metals (e.g. Al^3+^ and Cd^2+^) (Dai et al., 2021) and pathogenic bacteria (e.g. bacterial wilt) (Shen et al., 2018), resulting in the improvement of resistant taxa. This was also a self-protection mechanism of microorganisms against environmental stress. These were in accordance with results of Chen et al. (2022), in which probiotics *Bacteroidetes* and *Firmicutes* phyla were higher in acid soil than in non-acid soil. Additionally, *Acidobacteria* played an important role in the degradation of various organic materials and in the biogeosmic cycling of C, H and Fe (Kalam et al., 2020). And *Chloroflexi* is associated with nitrification and degradation of cellulose and polysaccharide (Sorokin et al., 2012). Both two phyla involve in C and N metabolism and play a key role in microbial community formation and stability under adverse environmental conditions. In brief, compared with LK, HK favored the growth of predicted copiotrophic groups and beneficial groups involved in pathogens and heavy metal resistance and N fixation, dissolution of P and K, while had negative with some oligotrophic taxa related to C, N metabolism. And the specific functions of potential beneficial bacteria (*Gemmatimonadetes* phyla and *Bacillus* and *Sphingomonas* genera) under environmental stress in tobacco-planting soils need to be investigated in the further research.

Different functional pathways could lead to different physiological consequences. In contrast to LK, HK had higher abundances of functional pathways mainly involved in carbohydrate metabolism (L2), such as starch and sucrose metabolism, glycolysis/gluconeogenesis and the TCA cycle (**Figure 7**). All the three pathways can release a large amount of energy for microbial life activities (Wang et al., 2015; Zhang et al., 2018). The TCA cycle is also the final metabolic pathway and hub of the three major nutrients (sugars, lipids, and amino acids). Some intermediate products formed during the decomposition of sucrose and starch can also be used as raw materials for the synthesis of biological macromolecules such as lipids, proteins and nucleic acids (Wang et al., 2015). In our study, pathways of homologous recombination, DNA replication, base excision repair and aminoacyl-tRNA biosynthesis, belonging to genetic information processing (L1) were also up-regulated in HK compared to LK (**Figure 7**). Homologous recombination plays an important role in the processing, integration and transformation of genes and is an important factor in maintaining gene frequency and gene diversity (Wielgoss et al., 2016). DNA replication enables genetic information to be passed from parent to offspring, thus ensuring the continuity of genetic information (Aklilu et al., 2014). Base excision repair is an important DNA oxidative damage defense response (Bauer et al., 2015). The primary function of aminoacyl-tRNA biosynthesis is protein synthesis, but they also play a role in gene expression, cell wall formation, protein labeling for degradation, and antibiotic biogenesis (Ling et al., 2009). Additionally, only three pathways of valine, leucine and isoleucine biosynthesis, pantothenate and CoA biosynthesis and C5-branched dibasic acid metabolism were enhanced in the soil of LK. They belonged to amino acid metabolism, metabolism of cofactors and vitamin and carbohydrate metabolism, respectively (**Figure 7**). These results indicated that, compared with LK, the addition of HK could provide more energy, promote microbial metabolism, and improve the functions of gene replication, recombination and repair. Our study also showed that MK promoted the functional pathways of one carbon pool by folate, streptomycin biosynthesis, monobactam biosynthesis and homologous recombination compared to LK (**Figure 7**). *Streptomycin* and *monobactam* are both antibiotics that effectively prevent disease (Li et al., 2017; Westhoff et al., 2019). One pool of carbon by folate is involved in protein synthesis and cell division (Ducker and Rabinowitz, 2017). Homologous recombination plays an important role in DNA damage repair and mutation processes (Wielgoss et al., 2016). The pathways of glyoxylate and dicarboxylate metabolism, alanine, aspartate and glutamate metabolism, RNA degradation and pantothenate and CoA biosynthesis were depleted in MK (**Figure 7**), which were related to sugar, fat and amino acid metabolism and cell regulation (Hjorth et al., 2006; Rubio et al., 2008). It was indicated that MK had both positive and negative effects on soil functions. All in all, we speculate that these pathways in microbes may have implications for plant survival and acid tolerance to some extent, which need further research.

### Soil properties shaped compositions of bacterial community and pathways

Previous studies reported that soil physicochemical properties played an important role in regulating microbial communities and functions (Lu et al., 2020; Kang et al., 2021). Our study showed that pH significantly shaped soil microbial community and functional composition. This result was consistent with many previous studies showing that pH was the most important indicator for determining bacterial composition, which was due to the relatively narrow growth tolerances of most bacterial taxa (Zhou et al., 2015). In this study, AK also significantly affected microbial community and KEGG pathways. The probable reason was that, excessive AK in the soil can affect the availability of other elements (e.g. Ca and N) (Nieder et al., 2011), thereby indirectly affecting the microbial community and function. Additionally, AS was another key factor in soil, which was significantly negatively correlated with multiple taxa and pathways (**Figure 9**). This was mainly attributable to damage to microorganisms caused by excess S in soil (Ma et al., 2020).

In summary, these results are broadly consistent with our hypothesis and advance our understanding of the impacts of K_2_SO_4_ application on the physicochemical and microbial properties of soils in typical tobacco fields, with implications for the scientific and reasonable application of K fertilizer in tobacco production. The common K_2_SO_4_ rate of 495 kg hm^-2^ in tobacco planting areas with *Alfisols* risks exacerbating soil acidification and adversely affecting plant growth. Considering yield, efficiency and environment, the optimal K_2_SO_4_ rate was 165-330 kg hm^-2^. Moreover, calcium magnesium phosphate fertilizer or organic fertilizer should also be used to prevent soil acidification.

## Conclusions

The present data showed that, compared with LK, HK promoted the N, K, S and OM contents of soil, while increased soil acidification. Due to this change, addition of HK resulted in higher percentages of predicted copiotrophic groups and beneficial bacterium and lower percentages of some oligotrophic taxa. According to the changes of KEGG pathways, carbohydrate metabolism and genetic information processing might improve in soils treated with HK. Additionally, the responses of soil physicochemical properties, composition of microbial community and functions to MK were less sensitive than HK. These results provide critical information to support the rational application of K fertilization in tobacco planting. However, further studies are required to investigate the variations of soil basic cations, heavy metal element, pathogenic bacteria and functional genes (C, N, P and S) to comprehensively and deeply evaluate the effect of excessive application of K_2_SO_4_ on ecological environment of tobacco-planting soil.

## Data availability statement

The datasets presented in this study can be found in online repositories. The names of the repository/repositories and accession number(s) can be found below: https://www.ncbi.nlm.nih.gov/, PRJNA805374.

## Author contributions

JD and WS conceived and designed the experiments. SK carried out the experiments. LT analyzed the data. YLi finalized the figures and tables. YLu and PC wrote the manuscript. All authors contributed to the article and approved the submitted version. 

## Funding

This work was financially supported by the Central Public-interest Scientific Institution Basal Research Fund (1610232022007), the Natural Science Foundation of Shandong Province (ZR2021QD036), Qingdao postdoctoral Foundation (2021).

## Conflict of interest

The authors declare that the research was conducted in the absence of any commercial or financial relationships that could be construed as a potential conflict of interest.

## Publisher’s note

All claims expressed in this article are solely those of the authors and do not necessarily represent those of their affiliated organizations, or those of the publisher, the editors and the reviewers. Any product that may be evaluated in this article, or claim that may be made by its manufacturer, is not guaranteed or endorsed by the publisher.
