# Supplementary material for: Long-term excessive application of K2SO4 fertilizer alters bacterial community and functional pathway of tobacco-planting soil
Source: Front Plant Sci. 2022 Sep 28;13:1005303. doi: 10.3389/fpls.2022.1005303 (PMC9554487; doi:10.3389/fpls.2022.1005303)
Supplement: Supplementary file 1 [file DataSheet_1.docx]

Table 1S Definition of OTU related to network

| OTU number | Phylum | OTU number | Phylum |
| --- | --- | --- | --- |
| OTU_69 | *Proteobacteria* | OTU_2452 | *Acidobacteria* |
| OTU_319 | *Proteobacteria* | OTU_2477 | *Acidobacteria* |
| OTU_350 | *Proteobacteria* | OTU_2532 | *Proteobacteria* |
| OTU_352 | *Firmicutes* | OTU_2600 | *Acidobacteria* |
| OTU_354 | *Proteobacteria* | OTU_2601 | *Proteobacteria* |
| OTU_357 | *Proteobacteria* | OTU_2682 | *Acidobacteria* |
| OTU_365 | *Proteobacteria* | OTU_2707 | *Proteobacteria* |
| OTU_391 | *Proteobacteria* | OTU_2709 | *Acidobacteria* |
| OTU_1001 | *Actinobacteria* | OTU_2710 | *Chloroflex* |
| OTU_1619 | *Gemmatimonadetes* | OTU_2711 | *Acidobacteria* |
| OTU_1622 | *Nitrospirae* | OTU_2713 | *Proteobacteria* |
| OTU_1858 | *Chloroflexi* | OTU_2715 | *Proteobacteria* |
| OTU_1860 | *Actinobacteria* | OTU_2717 | *Proteobacteria* |
| OTU_1929 | *Actinobacteria* | OTU_2721 | *Proteobacteria* |
| OTU_1934 | *Actinobacteria* | OTU_2733 | *Proteobacteria* |
| OTU_1936 | *Actinobacteria* | OTU_2734 | *Acidobacteria* |
| OTU_1938 | *Actinobacteria* | OTU_2735 | *Proteobacteria* |
| OTU_1939 | *Actinobacteria* | OTU_2735 | *Proteobacteria* |
| OTU_2069 | *Chloroflexi* | OTU_2752 | *Acidobacteria* |
| OTU_2094 | *Acidobacteria* | OTU_2757 | *Proteobacteria* |
| OTU_2296 | *Proteobacteria* | OTU_2763 | *Proteobacteria* |
| OTU_2313 | *Patescibacteria* | OTU_2795 | *Acidobacteria* |
| OTU_2323 | *Acidobacteria* | OTU_2874 | *Proteobacteria* |
| OTU_2448 | *Chloroflexi* | OTU_3170 | *Proteobacteria* |

Table 2S Definition of ko related to network

| Ko number | Level1 | Level2 | Level3 |
| --- | --- | --- | --- |
| ko00052 | Metabolism | Carbohydrate metabolism | Galactose metabolism |
| ko00130 | Metabolism | Metabolism of cofactors and vitamins | Ubiquinone and other terpenoid-quinone biosynthesis |
| ko00230 | Metabolism | Nucleotide metabolism | Purine metabolism |
| ko00240 | Metabolism | Nucleotide metabolism | Pyrimidine metabolism |
| ko00260 | Metabolism | Amino acid metabolism | Glycine, serine and threonine metabolism |
| ko00270 | Metabolism | Amino acid metabolism | Pyrimidine metabolism |
| ko00290 | Metabolism | Amino acid metabolism | Valine, leucine and isoleucine biosynthesis |
| ko00330 | Metabolism | Amino acid metabolism | Arginine and proline metabolism |
| ko00362 | Metabolism | Xenobiotics biodegradation and metabolism | Benzoate degradation |
| ko00364 | Metabolism | Xenobiotics biodegradation and metabolism | Fluorobenzoate degradation |
| ko00480 | Metabolism | Metabolism of other amino acids | Glutathione metabolism |
| ko00511 | Metabolism | Glycan biosynthesis and metabolism | Other glycan degradation |
| ko00520 | Metabolism | Carbohydrate metabolism | Amino sugar and nucleotide sugar metabolism |
| ko00531 | Metabolism | Glycan biosynthesis and metabolism | Glycosaminoglycan degradation |
| ko00564 | Metabolism | Lipid metabolism | Glycerophospholipid metabolism |
| ko00650 | Metabolism | Carbohydrate metabolism | Butanoate metabolism |
| ko00660 | Metabolism | Carbohydrate metabolism | C5-Branched dibasic acid metabolism |
| ko00670 | Metabolism | Metabolism of cofactors and vitamins | One carbon pool by folate |
| ko00730 | Metabolism | Metabolism of cofactors and vitamins | Thiamine metabolism |
| ko00760 | Metabolism | Metabolism of cofactors and vitamins | Nicotinate and nicotinamide metabolism |
| ko00770 | Metabolism | Metabolism of cofactors and vitamins | Pantothenate and CoA biosynthesis |
| ko00860 | Metabolism | Metabolism of cofactors and vitamins | Porphyrin and chlorophyll metabolism |
| ko00900 | Metabolism | Metabolism of terpenoids and polyketides | Terpenoid backbone biosynthesis |
| ko00920 | Metabolism | Energy metabolism | Sulfur metabolism |
| ko00930 | Metabolism | Xenobiotics biodegradation and metabolism | Caprolactam degradation |
| ko00940 | Metabolism | Biosynthesis of other secondary metabolites | Phenylpropanoid biosynthesis |
| ko00970 | Genetic Information Processing | Translation | Aminoacyl-tRNA biosynthesis |
| ko01503 | Human Diseases | Drug resistance: antimicrobial | Cationic antimicrobial peptide resistance |
| ko02010 | Environmental Information Processing | Membrane transport | ABC transporters |
| ko02020 | Environmental Information Processing | Signal transduction | Two-component system |
| ko02024 | Cellular Processes | Cellular community - prokaryotes | Quorum sensing |
| ko03010 | Genetic Information Processing | Translation | Ribosome |
| ko03018 | Genetic Information Processing | Folding, sorting and degradation | RNA degradation |
| ko03060 | Genetic Information Processing | Folding, sorting and degradation | Protein export |
| ko03070 | Environmental Information Processing | Membrane transport | Bacterial secretion system |
| ko03320 | Organismal Systems | Endocrine system | PPAR signaling pathway |
| ko03440 | Genetic Information Processing | Replication and repair | Homologous recombination |
| ko03450 | Genetic Information Processing | Replication and repair | Non-homologous end-joining |
| ko04146 | Cellular Processes | Transport and catabolism | Peroxisome |

Table 3S. The abundances of some of the copiotrophic families in *proteobacteria* (%)

| Treatments | *Burkholderiaceae* | *Rhodospirillaceae* | *Rhizobiaceae* |
| --- | --- | --- | --- |
| LK | 1.91±0.19b | 0.09±0.02b | 0.28±0.06b |
| MK | 2.01±0.15ab | 0.1±0.02b | 0.3±0.09b |
| HK | 2.66±0.32a | 0.18±0.02a | 0.51±0.06a |

Low amount of K_2_SO_4_; MK, Middle amount of K_2_SO_4_; HK, High amount of K_2_SO_4_. Different Lowercase letters in the same column indicate a significant difference of 0.05 between treatments
